# Parvovirus b19-Induced Acute Hepatitis With Hepatosplenomegaly and Polyarthropathy

**DOI:** 10.7759/cureus.21494

**Published:** 2022-01-22

**Authors:** Ubaid Khan, Rana Uzair Ahmad, Zabeeh Ullah, Tooba Fida, Muhammad Shehryar

**Affiliations:** 1 Department of Medicine, Mayo Hospital, Lahore, PAK; 2 Department of Internal Medicine, Mayo Hospital, Lahore, PAK; 3 Division of Research and Academic Affairs, Larkin Community Hospital Palm Springs Campus, Hialeah, USA; 4 Department of Cardiology, Mayo Hospital, Lahore, PAK

**Keywords:** hepatosplenomegaly, parovirus induced hepatitis, polyarthropathy, acute hepatitis, parvovirus b19

## Abstract

Parvovirus B19 infection can present with myriads of clinical diseases and syndromes; hepatitis and polyarthropathy are a few of these examples. Parvovirus frequently affects children but this condition has also been reported in adults.

The present case report discusses a case of a 43-year-old female who presented to the outpatient department (OPD) with complaints of high-grade fever and pain in multiple joints of her body for three days.

On examination, stiffness and swelling of the hand, knees, wrist, and ankles were noted. Laboratory investigations showed highly elevated aspartate transaminase (AST), alanine transaminase (ALT), and bilirubin. Electrocardiogram (ECG) and echocardiogram (ECHO) findings were unremarkable. PCR testing showed the presence of parvovirus. Parvovirus B19 infection led to the development of acute hepatitis, which appeared as yellowing of skin (jaundice) and led to hepatosplenomegaly. Parvovirus-induced polyarthropathy was also observed in the patient. The patient was managed with a parenteral course of ceftriaxone, paracetamol, and a normal saline infusion. Anti-viral drugs were also prescribed to the patient including ribavirin and pegylated interferon. This case study will explore how the patient was diagnosed and managed with conventional therapy and anti-viral to relieve parvovirus-induced hepatitis with hepatosplenomegaly and polyarthritis.

Acute hepatitis can be caused by viruses and other noninfectious causes, all of which must be cleared out to avoid chronic disease development. When patients present with joint pain and skin rashes, a thorough workup for viral indicators, medication histories, autoimmune and metabolic illnesses, and parvovirus b19 infection is required.

## Introduction

Parvoviruses are the smallest of deoxyribonucleic acid (DNA) viruses belonging to the Parvoviridae family, and human parvovirus b19 is the only parvovirus known to be pathogenic for humans [1]. Parvovirus b19 infection leads to a wide variety of symptoms in children as well as adults. Fever, headache, erythema infectiosum, and anemia are commonly observed symptoms in children while adults commonly suffer from multiple-joint pain and inflammation as well as generalized body aches. Thrombocytopenia and neutropenia are less commonly observed [2]. Our case represents a patient who developed polyarthropathy, rashes, and some rarely observed symptoms like acute hepatitis, which subsequently led to hepatosplenomegaly.

## Case presentation

A 43-year-old female presented to the outpatient department (OPD) with complaints of high-grade fever and pain in multiple joints of her body for three days. The patient was feeling pain in her wrists, hands, ankles, and knees. Initially, the pain was mild, and there were no associated symptoms. Three days later, the same patient came to OPD again with a complaint of the development of a rash on her both lower limbs. The rash only appeared just above both ankles up to the mid legs. This time patient was feeling excruciating pain in her multiple joints.

Besides rash and severe pain, the patient also complained about abdominal pain with nausea, anorexia, and mild discomfort. And she noticed yellow discoloration of skin (jaundice) 24 hours prior to the consultation, which was later confirmed by high level of bilirubin mentioned in Table 1. There were no other complains about any muscle pain, body pain, back pain, chest pain, cough, or breathing difficulties. Her vision was perfectly fine. There was no associated history of traveling or gastrointestinal/genitourinary infection. The patient denied alcohol and any intravenous drug intake. Detailed findings on examinations are mentioned in Table 2.

**Table 1:** Liver function tests AST, aspartate transaminase; ALT, alanine transaminase

Test	Value
AST	1700 units/l
ALT	1900 IU/l
Bilirubin	5.5 mg/dl

**Table 2:** Detailed systemic examination

Targeted system	Findings
General physical examination	Pulse rate 86/minute, respiratory rate 26/minute, temperature 39.1 C, blood pressure 135/82 mmHg. Marked yellow discoloration of skin and lower conjunctiva, no lymphadenopathy
Cardiovascular system	Fixed splitting second heart sound
Respiratory system	Normal respiratory sounds, normal chest
Integumentary system	No edema, pinkish erythematic maculopapular rash above both ankles up to the middle of legs. Pale/yellowish
Central nervous system	Reflexes were normal. None of the cranial nerves were affected. Normal motor function. Normal gait
Gastrointestinal system	Hepatomegaly and splenomegaly. Left lobe of liver was palpable and was nontender
Musculoskeletal system	Stiffness of knees, wrists, ankles, and hands. No Visible joint effusion. Swelling and tenderness. Inflamed synovial sheaths of fingers. Painful restricted movements of ankles, wrists, knees, and hands

Investigations

Lab investigations were ordered to confirm the diagnosis. CBC report is given in Table 3.

**Table 3:** CBC report CBC, complete blood count

Test	Value
Hemoglobin	13 g/dl dropped to 10.6 g/dl
Platelets	196,000 mm^3^
Mean corpuscular hemoglobin	25 pg/cell
Mean corpuscular volume	83 fl
Erythrocyte sedimentation rate	70 mm/h
Red blood cells	4-6 mm^2^
White blood cells	6000/ul

Blood culture was negative, and urine and stool cultures were found unremarkable. ECG and ECHO findings were normal. Similarly, on X-rays of hands, ankles, and feet no joint erosion was observed. However, there was visible joint effusion. On her second visit to OPD, the patient was asked to take a PCR test to detect parvovirus. PCR report came out positive. However, antiviral markers for detection of hepatitis B and hepatitis C, HBsAg, IgM anti-HB core antibody, anti-HBs, and anti-HBs, all were found negative. Parvovirus B19 IgM and parvovirus B19 IgG were also decreased over time (Table 4). 

**Table 4:** Other important findings CRP, C-reactive protein; LDH, lactate dehydrogenase; ANA, anti-nuclear antibody; RF, rheumatoid factor; CCP, cyclic citrullinated peptide

Test	Value
Urea	3.6 mg/dl
Serum creatinine	5 mg/dl
CRP	125 mg/l
LDH	448 units/l
ANA	Negative
RF	Negative
Anti-CCP	Negative
Serum ferritin	100.4 ng/dl

Diagnosis and management

From the above-given reports, it was clear that the patient was suffering from parvovirus-induced hepatitis as the patient had developed jaundice and there were associated symptoms of abdominal pain, anorexia, and nausea. Similarly, aspartate transaminase (AST) and alanine transaminase (ALT) were highly raised and PCR test came out positive for parvovirus. This verified the ultimate diagnosis as parvovirus-induced seronegative symmetrical polyarthropathy and acute hepatitis. The patient was in close contact with young primary school children, who could have been suffering from parvovirus itself but also were capable of transmitting it.

As a result of this diagnosis, the patient's first-care strategy included a parenteral course of ceftriaxone, paracetamol, and a normal saline infusion. Additionally, the methylprednisolone 1000 mg was given to the patient in pulses for five consecutive days. Antiviral medications were also prescribed to the patient including ribavirin and pegylated interferon. As expected, the outcomes were dramatic and favorable, resulting in a major improvement in the patient's symptoms.

## Discussion

Human parvovirus, a member of the Parvoviridae family, is a nonenveloped DNA virus. Its main mode of transmission is through respiratory droplets but can also spread through blood transfusions [3] and the placental route [4]. Parvovirus B19 predominantly affects school-going children, and less frequent manifestations are seen in pregnant women, immunocompromised individuals of all ages, and people who suffer from different types of hematological disorders.

Depending upon the age, condition of the immune system, and hematologic status, different people have a varying degree of clinical presentations. Most of the infected people go unnoticed as they are asymptomatic, and some patients show mild symptoms like fever, body rashes, and joint pain. In the acute phase, children suffer from erythema infectiosum while adults show arthritis and arthralgia. Chronic patients can undergo aplastic crisis and immunocompromised individuals face a chronic deficiency of red blood cells. Some cases of hepatitis have also been reported in the literature [5,6].

Our reported patient suffered from polyarthropathy, diffuse rashes over the lower limbs, and acute hepatitis in the initial phase; now the patient has developed hepatosplenomegaly, a very rare manifestation of parvovirus infection. IgM and IgG are detected in the acute phase, their concentration decreases over time, and some patients even lack antibodies against parvovirus b19 when the infection persists over a longer period of time [7].

## Conclusions

Acute hepatitis can have multiple infectious and noninfectious causes, and all of them must be ruled out to avoid the danger of leading to chronic diseases. Proper workup for viral markers, drug history, and autoimmune and metabolic diseases is necessary along with parvovirus b19 infection especially when patient suffers from joint pain and skin rashes. IgM and IgG are decreased over the period of time. it is imperative to test for parvovirus b19 through PCR levels. Follow-up and guidance for these patients are essential to save them from deadly chronic illnesses.
